# Sequential transcriptional programs underpin activation of hippocampal stem cells

**DOI:** 10.1126/sciadv.adu4523

**Published:** 2025-06-11

**Authors:** Piero Rigo, Sara Ahmed-de-Prado, Rebecca L. Johnston, Chandra Choudhury, François Guillemot, Lachlan Harris

**Affiliations:** ^1^ Neural Stem Cell Biology Laboratory, The Francis Crick Institute, London NW1 1AT, UK.; ^2^ QIMR Berghofer, Brisbane, QLD 4006, Australia.; ^3^ The University of Queensland, Brisbane, QLD 4067, Australia.; ^4^ Queensland University of Technology, Brisbane, QLD 4059, Australia.

## Abstract

Adult neural stem cells exist on a continuum from deep to shallow quiescence that changes in response to injury or aging; however, the transcription factors controlling these stepwise transitions have not been identified. Single-cell transcriptomic analyses of mice with loss of function or increased levels of the essential activation factor *Ascl1* reveal that *Ascl1* promotes the activation of hippocampal neural stem cells by driving these cells out of deep quiescence, despite its low protein expression in this state. Subsequently, during the transition from deep to shallow quiescence, *Ascl1* induces the expression of *Mycn*, which drives progression through shallow quiescent states toward a proliferating state. Together, these results define the required sequence of transcription factors during hippocampal neural stem cell activation and establish a combinatorial code for classifying these cells into deep and shallow quiescence.

## Teaser

The deep and shallow quiescent states of brain stem cells are controlled by distinct transcription factor proteins.

## INTRODUCTION

Stem cells are the source of all cells in the body and are highly active during embryonic development. After birth, adult stem cells, although few and less active, remain vitally important in maintaining the function of most organs through replacing damaged cells or by adding to the growth of tissue. To ensure a lifelong supply of adult tissue stem cells, many of these cells reside in a state of reversible growth arrest for prolonged periods, known as quiescence. These adult stem cells divide only when needed and frequently will return to quiescence after a small number of cell divisions, providing a long-term self-renewal capacity (*1*).

Cellular quiescence is not a single state but a spectrum. Stem cells that are in shallow quiescence, for example, in a G_alert_ state, have short activation times, whereas deeply quiescent cells require prolonged growth factor signaling to activate (*2*). The different states of quiescence have functional importance. For example, tissue injury pushes muscle stem cells and hematopoietic stem cells in uninjured areas of the body into a shallow quiescent state, thereby priming these cells for regenerative activity (*3*). In contrast, fasting pushes muscle stem cells into deep quiescence, decreasing regenerative capacity but increasing the resilience of the cells to stress (*4*).

Likewise, in the central nervous system, quiescent neural stem cells (NSCs) exhibit different depths of quiescence. Injury pushes quiescent NSCs in the subventricular zone into a “primed” state of shallow quiescence (*5*). NSCs that return to quiescence from a proliferating state enter a shallow “resting” state of quiescence, which increases the probability that these cells will reenter the cell cycle in the short term compared to cells that have not divided recently (*6*, *7*). Last, NSCs also progress into deeper states of quiescence the longer they remain quiescent, which reduces the activation rate of NSCs in aging animals (*7*–*9*).

Despite the functional impact of these different states, the transcription factors controlling how adult NSCs convert between states of deep and shallow quiescence have not been identified. Here, we reveal that, in adult mouse NSCs, activation from deep quiescence and shallow quiescence is governed by distinct and sequential molecular programs. Progression from deep quiescence to shallow quiescence is governed by the *Ascl1* transcription factor. ASCL1 induces the expression of *Mycn* to push cells through shallow states of quiescence into an active state. These defined transitions explain how populations of hippocampal NSCs are maintained in distinct states of quiescence.

## RESULTS

### Ascl1 deletion impairs progression from deep states of quiescence

Expression of the transcription factor ASCL1 is essential for the activation of adult hippocampal NSCs (AHNSCs) (*10*). ASCL1 must also be degraded for proliferating NSCs to return to a quiescent state (*11*). While analysis of ASCL1 function has focused so far on its role in the active NSCs, there is clear evidence that ASCL1 protein is also expressed in quiescent NSCs, albeit at lower levels, raising the possibility that it has a role in this population (*7*). To determine how ASCL1 might function during quiescence, we performed single-cell RNA-sequencing (scRNA-seq) of hippocampal NSCs after acute deletion of the *Ascl1* gene.

**Fig. 1.**
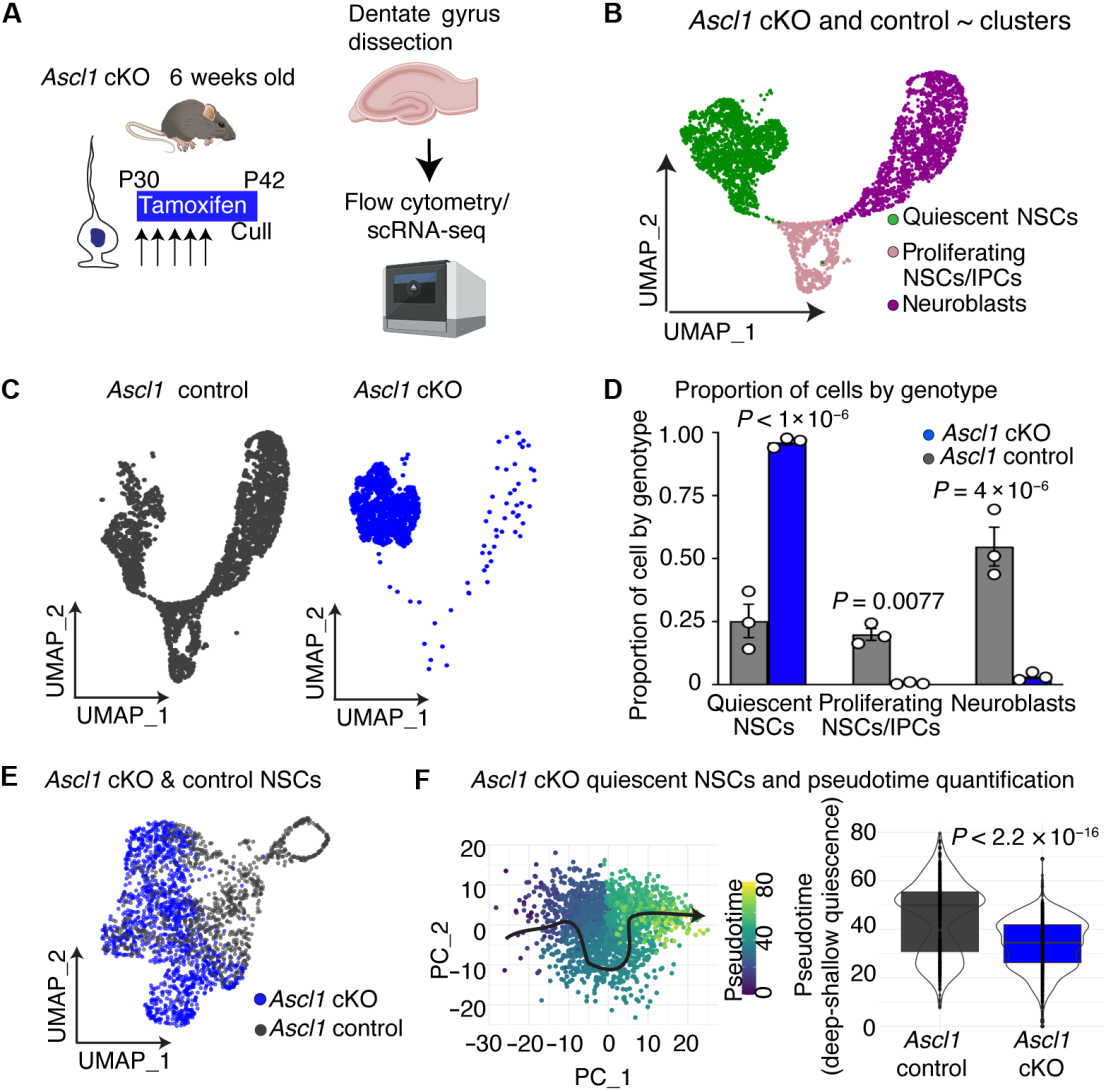
*Ascl1* loss reduces NSC activation by impeding progression from deep states of quiescence. (**A**) To assess how loss of ASCL1 affects quiescence, mouse hippocampal dentate gyrus was dissected, and genetically recombined cells (Glast-creERT2; RosaYFP) lacking *Ascl1* (*Ascl1* cKO) and controls were processed for scRNA-seq (*n* = 3 independent experiments). Created in BioRender. Harris, L. (2025) https://BioRender.com/u80z962. (**B**) UMAP of single-cell transcriptomes from *Ascl1* cKO and control mice showing quiescent NSCs, proliferating cells (NSCs and IPCs), and neuroblasts. (**C**) UMAP of single-cell transcriptomes from *Ascl1* cKO and control mice split by genotype. (**D**) Quantification of the proportion of quiescent NSCs, proliferating cells, and neuroblasts in *Ascl1* cKO and control mice. Dots indicate independent experiments. (**E**) UMAP of NSCs from *Ascl1* cKO and control mice. (**F**) Pseudotime analysis of quiescent NSCs from *Ascl1* cKO (1348 cells) and control mice (687 cells). Left: Analysis visualized using first two principal components (PCs), with cells colored by pseudotime and cell ordering indicated by black arrow. Right: Distribution of pseudotime values per genotype. Statistics: Kolmogorov-Smirnov test in (F) and multiple comparison *t* test in (D) reporting Holm-Šídák corrected *P* value.

We administered tamoxifen to Ascl1*fl/fl*; Glast-creERT2; Rosa-YFP mice [*Ascl1* conditional knockout (cKO)] at 1 month of age, deleting the *Ascl1* gene from NSCs and parenchymal astrocytes, while irreversibly labeling recombined cells with yellow fluorescent protein (YFP) (*10*, *12*, *13*). Twelve days after tamoxifen administration, we disassociated the dentate gyrus of *Ascl1* cKO mice, as well as control animals that lacked the conditional *Ascl1* allele. We sorted the recombined YFP^+^ *Ascl1* cKO and control cells by flow cytometry and performed scRNA-seq with the 10x Genomics platform (Fig. 1A and table S1). In total, 12,548 cells from *Ascl1* cKO mice and 9189 cells from controls across three independent experiments passed quality control. We first inspected our data by isolating the three main neurogenic cell clusters (Fig. 1B). These clusters comprised quiescent NSCs, proliferating cells (both proliferating NSCs and intermediate progenitor cells (IPCs), which cluster together due to dominance of cell-cycle gene expression), and neuroblasts, which we demarcated according to the expression of canonical markers *Hopx*-high/*S100b*-low (*14*), *Eomes/Mki67*, and *Dcx* (*15*), respectively, as previously described (*7*). Consistent with the established role for ASCL1 in active NSCs (*10*), loss of ASCL1 led to an almost complete absence of actively dividing cells in *Ascl1* cKO mice (Fig. 1C). In our scRNA-seq data of *Ascl1* cKO mice, only 0.55% of cells were actively proliferating compared to 20% of control cells (Fig. 1D; *P* = 0.0077). We have previously reported that this block in proliferation leads to an accumulation of NSCs in a quiescent state due to a lack of division-coupled depletion (*10*, *16*). In our scRNA-seq data 96.2% of cells were quiescent NSCs compared to 25.3% of control cells (Fig. 1D; *P* < 1 × 10^−6^). Last, the block in proliferation also leads to a histological loss of immature neuronal progeny (*10*), and, in our scRNA-seq data, just 3.27% of cells in *Ascl1* cKO mice were neuroblasts compared to 54.8% of controls cells (Fig. 1D; *P* = 4 × 10^−6^). The small number of aberrant proliferating cells and neuroblasts in our scRNA-seq data from *Ascl1* cKO mice was likely due to incomplete recombination events (*10*), as we detected *Ascl1* mRNA expression in most of proliferating cells in the *Ascl1* cKO dataset. Our scRNA-seq data, therefore, faithfully recapitulates the histological phenotype of *Ascl1* cKO animals (*10*), allowing us to use this sequencing tool to determine how *Ascl1* loss affects the underlying biology of the quiescent NSC population.

To examine the effect of *Ascl1* deletion on the quiescent stem cell population, we re-clustered the scRNA-seq data to only include NSCs (Fig. 1E; see Materials and Methods). This analysis reinforced the finding that there were fewer proliferating NSCs in cKO mice compared to those in controls (fig. S1A; *P* = 0.0085) and a concomitant increase in the proportion of quiescent NSCs (*P* = 0.0085). We then re-clustered the data a final time to only include quiescent NSCs by removing proliferating cells (Fig. 1F). We arranged the quiescent NSCs along a pseudotime axis from deep to shallow quiescence using the trajectory inference tool Slingshot (Fig. 1F) (*17*). Loss of ASCL1 led to a pronounced phenotype in quiescent NSCs, where much of the NSC population resided in deep states of quiescence, as supported by a statistical difference in distribution between *Ascl1* cKO and control cells (*P* < 2.2 × 10^−16^). Specifically, 96% of quiescent NSCs from *Ascl1* cKO mice occupied the deepest 50% of pseudotime positions in control mice (Fig. 1F and fig. S1, B and G). These data demonstrate that loss of ASCL1 in adult NSCs causes profound transcriptional changes to all NSCs, including quiescent NSCs where it is expressed at low levels, ultimately moving the population into a deeper quiescent state or preventing deeply quiescent NSCs from moving into shallow quiescence. We next tested whether, conversely, increasing the level of ASCL1 protein might shift deeply quiescent NSCs to a shallow state.

### Huwe1 loss of function promotes progression from deep states of quiescence

**Fig. 2.**
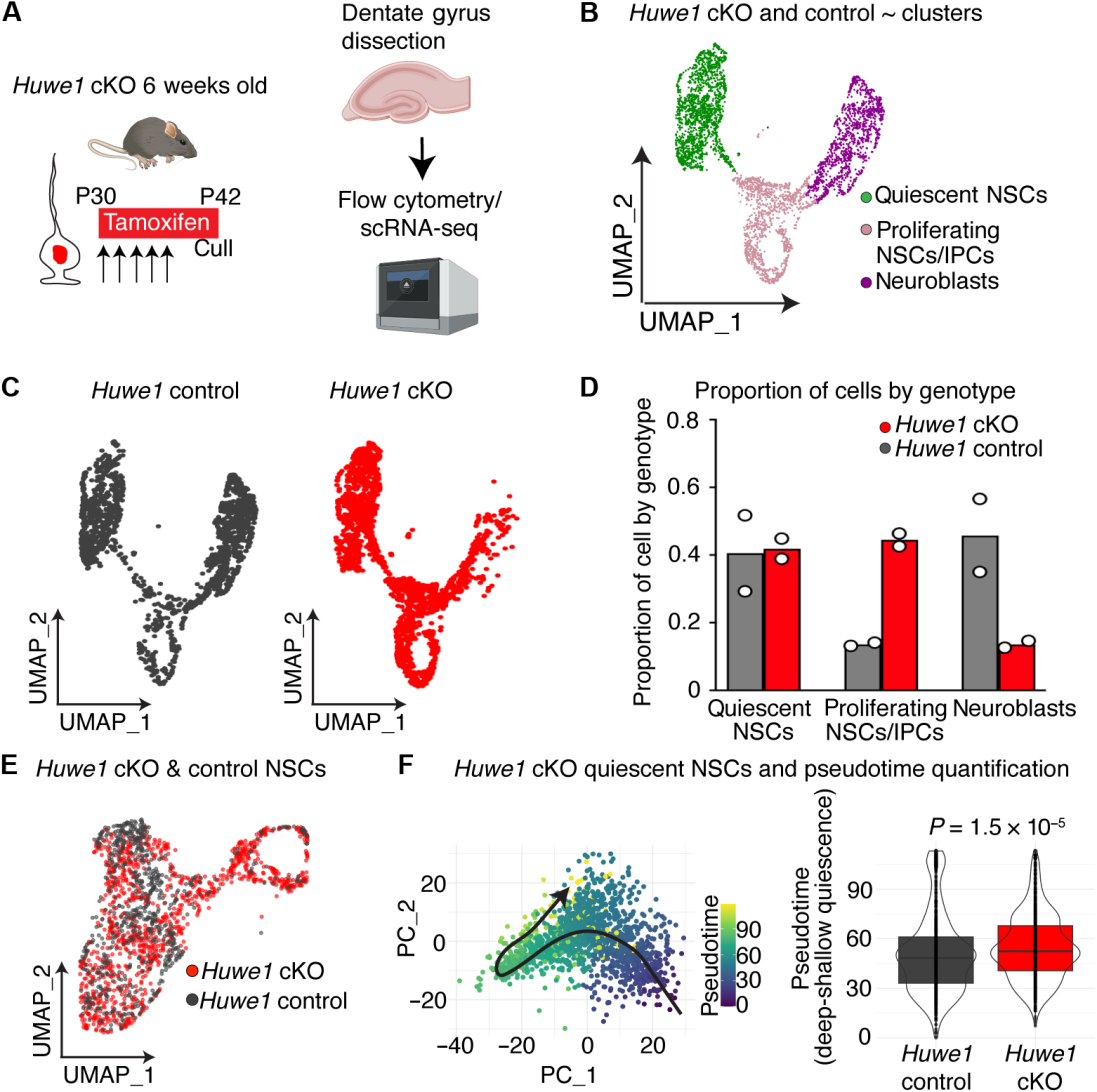
*Huwe1* loss of function increases NSC activation by promoting progression from deep states of quiescence. (**A**) To assess how loss of HUWE1 affects quiescence, mouse hippocampal dentate gyrus was dissected, and genetically recombined cells (Glast-creERT2; RosaYFP) lacking *Huwe1* (*Huwe1* cKO) and controls were processed for scRNA-seq. Created in BioRender. Harris, L. (2025) https://BioRender.com/u80z962. (**B**) UMAP of single-cell transcriptomes from *Huwe1* cKO and control mice showing quiescent NSCs, proliferating cells (NSCs and IPCs), and neuroblasts. (**C**) UMAP of single-cell transcriptomes from *Huwe1* cKO and control mice colored by genotype. (**D**) Quantification of the proportion of quiescent NSCs, proliferating cells, and neuroblasts in *Huwe1* cKO and control mice. Dots indicate independent experiments. (**E**) UMAP of NSCs from *Huwe1* cKO and control mice. (**F**) Pseudotime analysis of quiescent NSCs from *Huwe1* cKO (860 cells) and control mice (671 cells). Left: Analysis visualized using first two principal components, with cells colored by pseudotime and cell ordering indicated by black arrow. Right: Distribution of pseudotime values per genotype. Statistics: Kolmogorov-Smirnov test in (F).

To examine the effect of increasing ASCL1 levels, we repeated the scRNA-seq experimental design, except this time deleting the gene encoding the ubiquitin-ligase HUWE1 (Fig. 2A). The protein HUWE1 marks ASCL1 for proteasomal degradation. Our previous work has demonstrated that ASCL1 is a major substrate of HUWE1 in AHNSCs, and, thus, its deletion leads to elevated levels of ASCL1 protein in proliferating NSCs (*11*) and in quiescent NSCs (*7*). The scRNA-seq analysis of dentate gyrus cells isolated from two independent experiments of *Huwe1* cKO mice (6784 cells) and controls (5187 cells) recapitulated our previous immunolabeling characterization of this strain (*11*). Specifically, a larger percentage of cells from the neurogenic lineage were proliferating in *Huwe1* cKO mice (44.5% of cells) compared to those in control cells (13.6% of cells) (Fig. 2, B to D). Furthermore, as we have previously shown, *Huwe1* cKO neuroblasts fail to exit the cell cycle and differentiate, resulting in apoptosis (*11*). This phenotype was manifested in the scRNA-seq analysis as a reduction in the proportion of neuroblasts (Fig. 2, B to D). We then re-clustered the scRNA-seq data to only include NSCs (Fig. 2E), which reinforced the trend toward an increase in proliferating NSCs and a decrease in quiescent NSCs within cKO mice compared to that in control mice (fig. S1C). While sample size for the scRNA-seq experiments limits our ability to draw statistical conclusions from cell proportions alone, these trends were consistent with our previous immunostaining findings (*7*, *11*).

We then refined the dataset further by only retaining quiescent cells (Fig. 2F) and organized them into a pseudotime trajectory. We found that the increase in proliferation in *Huwe1* cKO mice was due to a marked shift in the stem cell population so that more mutant cells fell within the shallow end of the quiescence-to-activity trajectory and were likewise depleted from deep states of quiescence, as supported by a significant difference in distribution between *Huwe1* cKO and control cells (Fig. 2F; *P* = 1.5 × 10^−5^). Specifically, 23% more NSCs existed in shallow states of quiescence in *Huwe1* cKO mice relative to controls (fig. S1, D and G). Together, the pseudotime trajectories of *Ascl1–* and *Huwe1–*loss-of-function mice reveal an unexpectedly early role for ASCL1 in the activation process. Despite the low levels of ASCL1 protein in deeply quiescent NSCs, ASCL1 is required to promote the progression of these cells from deep to shallow quiescence. These results might help to explain our previous observations where more NSCs accumulated in deeper quiescent states with age, a phenomenon tightly correlated with reduced ASCL1 protein levels in quiescent hippocampal NSCs in older mice (*7*).

### Loss of three *Myc* genes from adult NSCs is comparable to loss of Mycn alone

We next wondered whether the subsequent progression of adult NSCs through shallow quiescence and into an proliferating state might be associated with the activity of transcription factor(s) other than ASCL1. NSCs in shallow quiescence such as primed and resting cells have higher expression of genes associated with mRNA transport and protein translation (*5*, *7*, *18*). These pathways have been shown to be up-regulated by *Myc* transcription factors in other quiescence-associated contexts, such as during diapause in preimplantation embryos and in dormant hematopoietic stem cells (*19*, *20*). This led us to hypothesize that the *Myc* gene family might similarly increase the expression of these pathways in quiescent NSCs to promote the activation of these cells out of shallow quiescence.

**Fig. 3.**
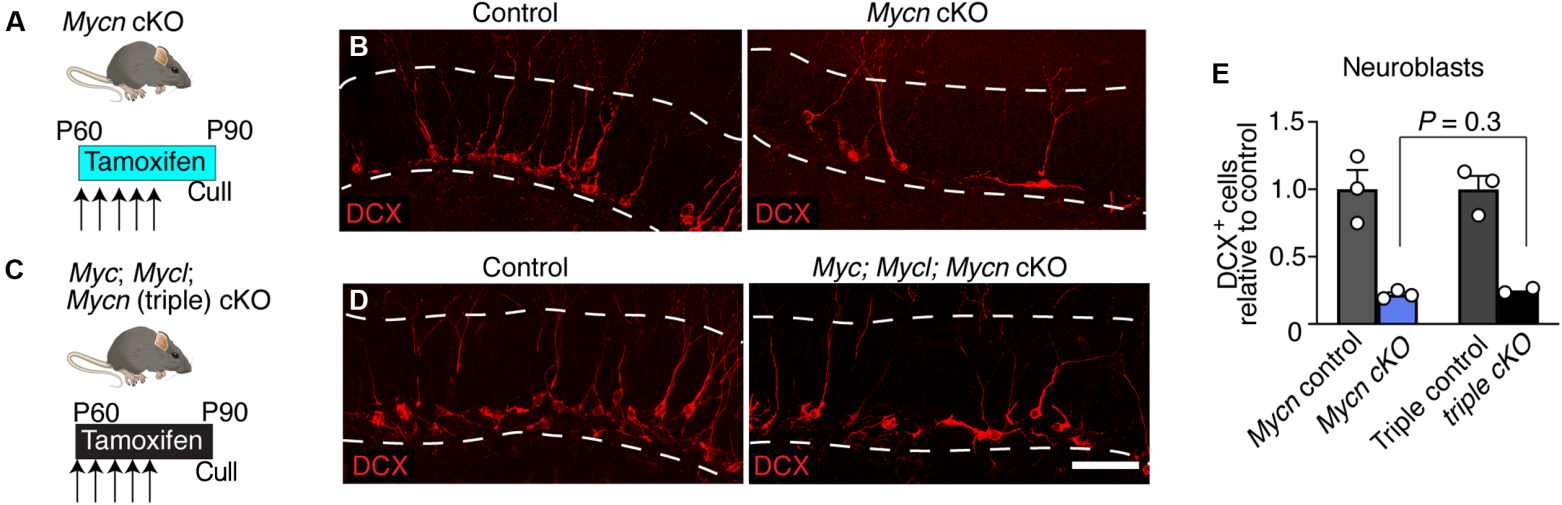
Loss of three *Myc* genes from adult NSCs is comparable to loss of *Mycn* alone. (**A**) *Mycn* cKO (*n* = 3) and control mice (*n* = 3) were injected with tamoxifen for 5 days and culled 30 days later. (**B**) DCX staining in *Mycn* cKO and control mice. (**C**) Triple *Myc* cKO (*n* = 2) and control mice (*n* = 3) were injected with tamoxifen for 5 days and culled 30 days later. (**D**) DCX staining in triple *Myc* cKO and control mice. (**E**) Fold change in neuroblast number in *Mycn* cKO and triple *Myc* cKO mice relative to respective controls. Graph in E shows means ± SEM. Statistics: Unpaired *t* test (E). Scale bar [located in (D)], 60 μm in (B) and (D). Dashed lines demarcate granule cell layer in (B) and (D).

We first individually deleted the three *Myc* gene family members from adult stem cells and parenchymal astrocytes by crossing conditional *Myc* (*21*), *Mycn* (*22*), and *Mycl* mice (*23*) to *Glast-creERT2* animals. For each strain, we administered tamoxifen to 2-month-old animals, culling them 30 days later (fig. S2). Potential genetic redundancy between *Myc* family members was explored by generating a triple cKO strain (Fig. 3) (*24*). We stained brain slices for the immature neuronal marker Doublecortin (DCX), as a preliminary screen for underlying defects in NSC activation, which, all else being equal, would be expected to culminate in reduced levels of adult neurogenesis. Of the three family members, *Mycn* had the highest mRNA expression in cells of the neurogenic lineage (fig. S2). Consistent with this, deletion of *Mycn* from adult NSCs, although not that of *Myc* or *Mycl*, led to a substantial reduction in DCX-positive cells compared to control mice (fig. S2). Conditional deletion of all three *Myc* genes did not exacerbate the phenotype beyond that of the single *Mycn* gene deletion (Fig. 3; *P* = 0.30). Therefore, *Mycn*, but not *Myc* or *Mycl*, has an important role in promoting adult neurogenesis in the mouse hippocampus.

### MYCN promotes progression from shallow states of quiescence

**Fig. 4.**
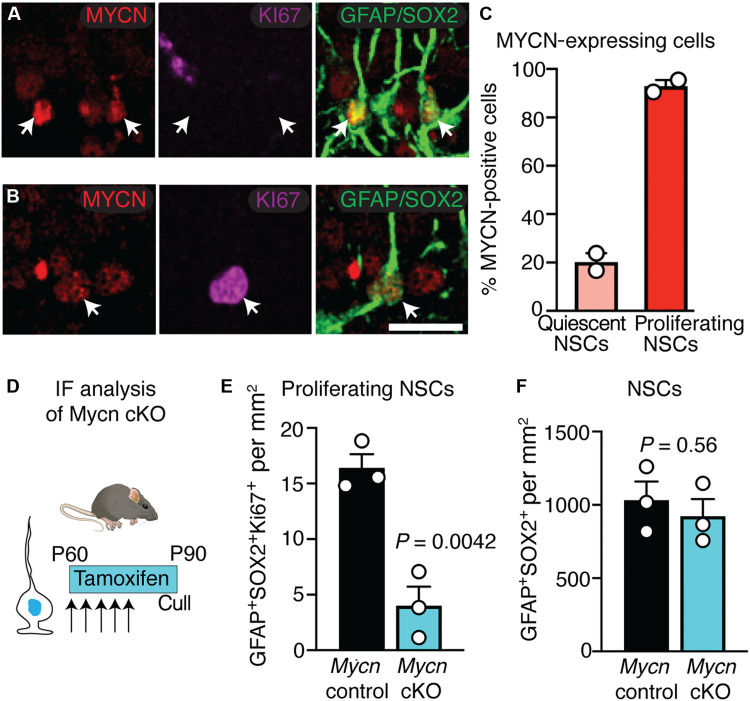
*Mycn* loss reduces the number of proliferating NSCs. (**A**) MYCN protein is detected in a minority of quiescent NSCs in the adult hippocampus. (**B**) MYCN protein is detected in a majority of proliferating NSCs in the adult hippocampus. (**C**) Quantification of the proportion of quiescent and proliferating NSCs expressing MYCN by cell-type (*n* = 2 mice). (**D**) Conditional, inducible deletion of *Mycn* (Glast-creERT2) followed by immunofluorescence (IF) analysis. *Mycn* cKO and control mice received tamoxifen for 5 days and were culled 30 days later. (**E**) Quantification of the number of proliferating NSCs in *Mycn* cKO mice (*n* = 3) and controls (*n* = 3). (**F**) Quantification of the number of NSCs in *Mycn* cKO mice (*n* = 3) and controls (*n* = 3). Graphs in (E) and (F) show means ± SEM. Statistics: Unpaired *t* test (E and F). Scale bar (located in B), 20 μm in (A) and (B).

We next examined whether the phenotype of *Mycn* cKO was brought about by an underlying impairment in the NSC activation process. The expression of MYCN protein supported a possible role in regulating NSC activation, with 20% of quiescent NSCs (radial GFAP^+^SOX2^+^ cells) expressing the protein in *Mycn* control mice at 6 weeks of age (Fig. 4, A to C), which largely reflected the mRNA expression in a subset of NSCs in shallow quiescence (fig. S2I). Quantification of NSCs revealed that deletion of *Mycn* reduced the proportion of AHNSCs that were proliferating by 80% (Fig. 4, D and E; *P* = 0.0042) but did not affect overall NSC number (Fig. 4F; *P* = 0.56).

**Fig. 5.**
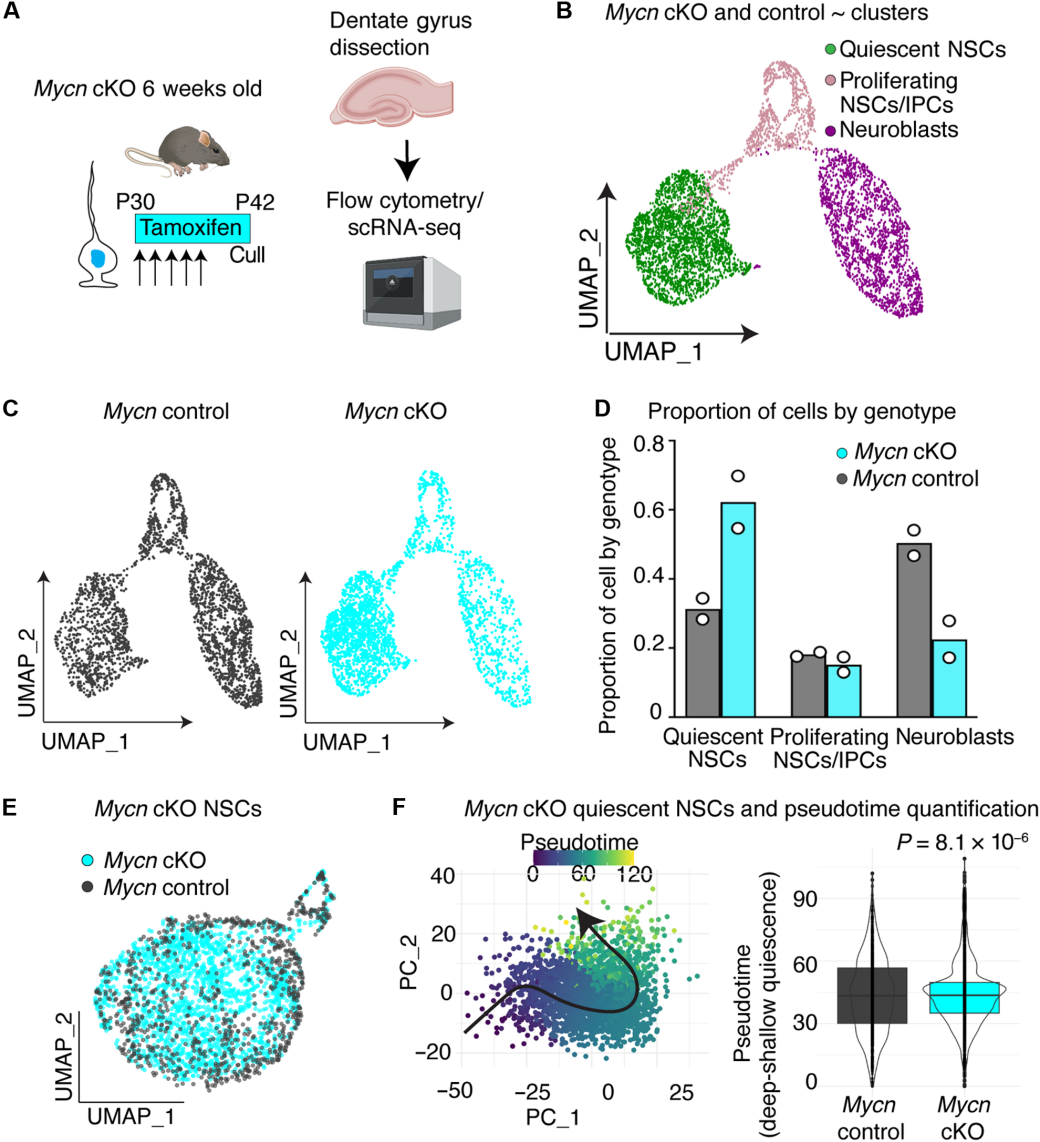
*Mycn* loss reduces NSC activation by impeding progression from shallow states of quiescence. (**A**) To assess how loss of MYCN affects NSC quiescence, mouse hippocampal dentate gyrus was dissected, and Nestin::GFP-positive cells from *Mycn* cKO and controls were processed for scRNA-seq. Created in BioRender. Harris, L. (2025) https://BioRender.com/u80z962. (**B**) UMAP of single-cell transcriptomes from *Mycn* cKO and control mice showing quiescent NSCs, proliferating cells (NSCs and IPCs), and neuroblasts. (**C**) UMAP of single-cell transcriptomes from *Mycn* cKO and control split by genotype. (**D**) Quantification of quiescent NSCs, proliferating cells, and neuroblasts in *Mycn* cKO and control mice. Dots indicate independent experiments. (**E**) UMAP of NSCs from *Mycn* cKO and control mice. (**F**) Pseudotime analysis of quiescent NSCs from *Mycn* cKO (1817 cells) and control mice (870 cells). Left: Analysis visualized using first two principal components, with cells colored by pseudotime and cell ordering indicated by black arrow. Right: Distribution of pseudotime values per genotype. Statistics: Kolmogorov-Smirnov test in (F).

To define how Mycn loss leads to decreased proportion of proliferating hippocampal NSCs, we performed scRNA-seq of *Mycn* cKO mice and controls, in a design that mirrored that of the *Ascl1* and *Huwe1* cKO experiments (Fig. 5A). In total, across two independent experimental replicates, we sequenced 9187 *Mycn* cKO cells and 6845 control cells. Consistent with our immunolabeling data (Fig. 4D), an increased percentage of cells from *Mycn* cKO mice were quiescent NSCs compared to those from controls (62.3% versus 31.4%), and there were also fewer neuroblasts (22.5% versus 50.4%) (Fig. 5, B to D). Given the limited sample size of the single-cell dataset, we report these findings as descriptive trends and avoid drawing statistical conclusions regarding differences in cell proportions.

To test how MYCN might affect NSC activation, we then re-clustered the scRNA-seq data to only include NSCs (Fig. 5E). This analysis supported the trend observed in our immunostaining data, with fewer proliferating NSCs in *Mycn* cKO mice compared to those in control mice (fig. S1E). We then further refined the dataset by isolating only quiescent cells and organized them into a pseudotime trajectory (Fig. 5F). Unlike NSCs from *Ascl1* cKO mice, which accumulated in deep states of quiescence, the *Mycn* cKO cells entirely overlapped with control cells. However, quiescent NSCs from *Mycn* cKO mice were not phenotypically normal, instead they demonstrated a distinct phenotype, whereupon they accumulated approximately halfway along the pseudotime trajectory, as supported by a statistical difference in distribution between *Mycn* cKO and control cells (Fig. 5F; *P* = 8.1 × 10^−6^). These data suggest that, while both *Ascl1* and *Mycn* deletion leads to a loss or decrease in stem cell activation, these phenotypes arise, at least, in part, through different cellular mechanisms. *Ascl1* loss principally hinders the ability of cells to leave deep quiescence, whereas loss of *Mycn* impedes progression through shallow quiescence (fig. S1G). Consistent with this, using tradeSeq (*25*, *26*), we found that the number of differentially expressed genes between *Ascl1 cKO* mice and controls was highest during early phases of pseudotime (deep quiescence, defined as cells positioned before the median pseudotime position of *Ascl1* control cells), while the number of differentially expressed genes in *Mycn* cKO mice and controls was highest in shallow quiescence (defined as cells positioned after the median pseudotime position of *Mycn* control cells) (fig. S3).

### Sequential Ascl1-Mycn programs govern NSC activation

**Fig. 6.**
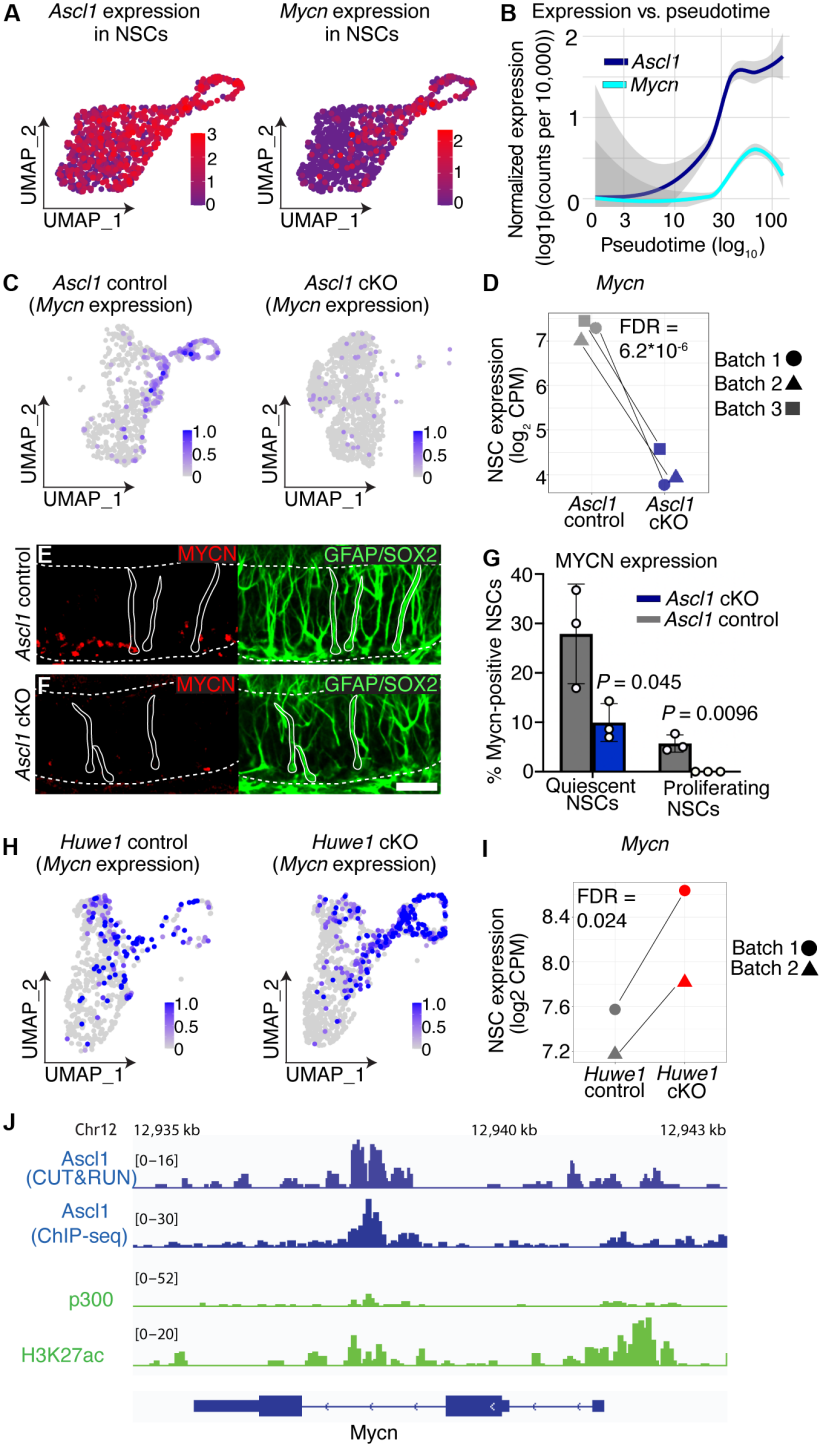
A sequential *Ascl1*-*Mycn* program drives NSC activation. (**A**) UMAP showing *Ascl1* and *Mycn* expression in wild-type NSCs. Data from *Ascl1* control mice in Fig. 1. (**B**) *Ascl1* and *Mycn* expression in wild-type NSCs along pseudotime from deep quiescence to a proliferating state. Shaded area indicates the 95% confidence interval. Data from *Ascl1* control mice in Fig. 1. (**C**) UMAP showing *Mycn* expression in *Ascl1* cKO and control NSCs. (**D**) Scatterplot of *Mycn* expression in *Ascl1* cKO and control NSCs. Batch indicates experiment number. (**E**) MYCN protein expression in *Ascl1* control NSCs at 6 weeks of age, 1 week after completing a 5-day course of daily tamoxifen gavage. (**F**) MYCN protein expression in *Ascl1* cKO NSCs at 6 weeks of age, 1 week after completing a 5-day course of tamoxifen gavage. Scale bar, 30 μm. (**G**) Quantification of the proportion of NSCs that are quiescent and positive for MYCN (MYCN^+^KI67^–^) and the proportion that are proliferating and positive for MYCN (MYCN^+^KI67^+^) in *Ascl1 cKO* (*n* = 3) and control mice (*n* = 3). (**H**) UMAP showing *Mycn* expression in *Huwe1* cKO and control NSCs. (**I**) Scatterplot of *Mycn* expression in *Huwe1* cKO and control NSCs. Batch indicates experiment number. (**J**) ASCL1 CUT&RUN data from adult hippocampus-derived NSCs (this study) and ChIP data from NS5 cells at *Mycn* locus (*10*, *27*). Statistics: Pseudo-bulk differential expression in (D) and (I) reporting FDR-corrected *P* value and *t* test in (G).

The roles of ASCL1 and MYCN in promoting progression toward an active state from deep and shallow states of quiescence, respectively, suggested that ASCL1 might induce the expression of MYCN during the activation process. Consistent with this, *Ascl1* mRNA levels increase markedly before the induction of *Mycn* mRNA, as cells progress from deep to shallow states of quiescence (Fig. 6, A and B, and fig. S4). To explore this relationship further, we performed differential expression analysis on NSCs from *Ascl1* cKO mice and controls using a pseudo-bulk approach (table S2). In NSCs from *Ascl1* cKO mice, *Mycn* was the second most strongly down-regulated transcription factor in the dataset ranked by *P* value [Fig. 6, C and D, and table S2; false discovery rate (FDR) = 6.15 × 10^−6^]. We verified that MYCN was also down-regulated at a protein level in quiescent NSCs (*P* = 0.045) and proliferating NSCs (*P =* 0.0096) from *Ascl1 cKO* mice compared to those from controls (Fig. 6, E to G). Similarly, we performed differential expression analysis of NSCs from *Huwe1* cKO mice and controls (table S3). In this analysis, loss of *Huwe1* led to a strong up-regulation of *Mycn* (Fig. 6, H and I, and FDR = 0.0241). Furthermore, we analyzed a previously published chromatin immunoprecipitation sequencing (ChIP-seq) dataset of ASCL1 binding in NS5 cells and found an ASCL1 peak overlapping with active enhancer marks P300 and H3K27ac in an intronic region of the *Mycn* gene (Fig. 6J) (*10*, *27*). We confirmed this peak by performing Cleavage Under Targets and Release Using Nuclease (CUT&RUN) on primary hippocampal NSC cultures (Fig. 6J). Together, these data demonstrate that ASCL1 promotes the expression of *Mycn* during hippocampal NSC activation, likely through direct transcriptional activation.

We next sought to compare the transcriptional programs controlled by *Ascl1* during deep quiescence and *Mycn* during shallow quiescence. To do this, we performed differential expression analysis between cKO and control only on the quiescent NSC fraction from both the *Ascl1* datasets (table S2; 158 differentially expressed genes) and *Mycn* datasets (table S4; 92 differentially expressed genes). Gene ontology (GO) enrichment analysis of biological process terms (table S2) revealed that loss of *Ascl1* led to enrichment of the notch signaling pathway (e.g., down-regulation of genes *Dll1*, *Notch1/*2, and *Hes5*) and positive regulation of cyclin-dependent protein serine/threonine kinase activity (e.g., down-regulation of *Ccnd1/2* and *Egfr* genes). Loss of *Ascl1* also led to enrichment of regulation of BMP signaling pathway, including decreased expression of BMP antagonist *Nbl1* and altered expression of genes associated with cell-substrate adhesion and mitochondrial membrane potential (*Ndufc2* and *Mfn1*). In contrast, loss of *Mycn* led to enriched GO terms relating to biosynthesis, specifically GO terms such as cytoplasmic translation (e.g., down-regulation of *Rpl29*, *Rps12*, and *Rps8* genes) and regulation of transcription from RNA polymerase II promoter in response to stress (e.g., *Egr1*, *Klf2*, and *Pppr1r15a*) (table S4). No ribosomal genes or mRNA processing pathways were enriched in the *Ascl1* dataset (table S2).

Furthermore, we mapped the down-regulated genes in these quiescent NSC datasets, i.e., those likely to be transcriptionally activated by *Ascl1* and *Mycn*, onto deep, shallow, and proliferating states in control cells. This analysis revealed distinct expression patterns: Genes down-regulated in *Ascl1* cKO mice showed a steady increase in expression from deep to shallow quiescence, whereas genes down-regulated in *Mycn* cKO mice exhibited increased expression only during the transition from shallow quiescence to the active state (fig. S4). These analyses demonstrate a clear separation of roles where ASCL1 promotes activation from deep quiescence by modulating the activity of key signaling pathways (e.g., cell adhesion and *BMP* signaling and metabolic modulation through mitochondrial membrane potential) (*28*–*30*), whereas MYCN promotes activation from shallow quiescence by increasing transcription and translation (*18*).

## DISCUSSION

Quiescence is not a uniform cellular state; rather, it exists as a continuous spectrum of varying degrees of depth. Transitions between deep and shallow states of quiescence have been associated with the degree of lysosomal activity and autophagic flux (*31*). However, outside of this, relatively little is known about the ordering of events during these transitions, including in the adult nervous system. Using scRNA-seq, we have demonstrated that these transitions can be separated into distinct phases in quiescent hippocampal NSCs, where sequential activation of ASCL1 and MYCN transcription factors leads to NSC activation.

Prior work has focused on the role of ASCL1 in proliferating NSCs (*10*, *11*), whereas our analysis has demonstrated a role for ASCL1 in quiescent NSCs. Specifically, our data demonstrates that ASCL1 is required to promote the progression of hippocampal NSCs from deep states to shallow states of quiescence. We have previously observed ASCL1 levels decline during aging, and this is correlated with an accumulation of hippocampal NSCs in deep states of quiescence (*7*). Our current data further strengthens the argument that this is a causal relationship. The role for ASCL1 in promoting activation from deep states of quiescence may appear unexpected because ASCL1 protein is typically only detected in proliferating NSCs when using conventional antibody staining techniques. However, despite being broadly transcribed, ASCL1 is typically only detected in proliferating NSCs because it undergoes proteasomal degradation due to destabilization by inhibitor of DNA binding transcription factor proteins in quiescent NSCs (*32*). Despite these post-translational mechanisms controlling ASCL1 levels in quiescent NSCs, it is clear that small amounts of protein are produced. For example, using sensitive antibodies against a VENUS fusion protein, we have previously detected ASCL1 protein in more than 50% of quiescent NSCs, at least in young mice (*7*). These findings reflect a report demonstrating that pancreatic epithelial progenitors express high levels of the mRNA encoding the basic helix-loop-helix transcription factor *Neurog3* but low levels of the NGN3 protein, which, nonetheless, play an important part in maintaining the progenitor state (*33*, *34*).

Conversely, we found that the *Mycn* transcription factor promotes activation from shallow states of quiescence. *Mycn* appears to be important in increasing biosynthesis, consistent with its role in other systems such as in embryonic diapause (*20*) or in quiescent hematopoietic stem cells (*19*). We analyzed conditional genetic knockout mice of *Mycn*, *Myc*, and *Mycl* side by side and only observed substantial defects in *Mycn* mice, and the phenotype of triple knockout mice was comparable to loss of *Mycn* alone. While we cannot rule out minor roles for *Myc/Mycl* in the activation process, *Myc* has recently been suggested to have a role in promoting hippocampal NSC activation (*35*), *Mycn* is the dominant family member in this context.

These sequential transcriptional programs governing progression from deep quiescence to shallow quiescence are likely linked through direct transcriptional regulation, where ASCL1 activates the expression of MYCN. ASCL1 has previously been shown to transcriptionally regulate MYCN in other scenarios. For example, MYCN is a target gene of ASCL1 in glioblastoma (*36*). Similarly, in neuroblastoma, ASCL1 acts as a pioneer factor where it then recruits and cooperates with MYCN at genomic loci to drive adrenergic cell fate conversion (*37*). Whether ASCL1 and MYCN also drive gene expression in a cooperative manner in quiescent NSCs is unclear, although relatively few genes were commonly mis-regulated between both datasets, which would argue for limited cooperation. Regardless of the exact functional relationship between these transcription factor proteins, a useful mRNA combinatorial code can be generated to classify hippocampal NSCs into deep states of quiescence (*Ascl1^+^Mycn-Ki67*^−^), shallow states of quiescence (*Ascl1^+^Mycn^+^Ki67*^−^), and proliferating states (*Ascl1^+^Mycn^+^Ki67^+^*). Future studies should investigate whether *Ascl1^+^Mycn^+^Ki67*^–^ hippocampal NSCs encompass all cells in shallow quiescence or whether populations such as resting NSCs (*7*), which emerge during aging, or primed NSCs, which arise in response to injury (*5*), represent separate populations.

### Limitations of study

MYCN also serves as a substrate for HUWE1-mediated ubiquitination during embryonic brain development (*38*). While our prior work established ASCL1 as the primary target of HUWE1 in hippocampal NSCs (*11*), the possibility that elevated MYCN protein levels also contribute to the phenotype observed in *Huwe1* cKO mice cannot be ruled out. Regardless, our conditional deletion of the *Mycn* gene demonstrates that the primary function of *Mycn* is to promote progression through shallow quiescence.

## MATERIALS AND METHODS

### Experimental design

The objectives of this study were to identify the functional effect of deleting *Ascl1* through scRNA-seq. Separately, we hypothesized that *Myc* genes might be important in regulating shallow states of quiescence and that loss of *Myc* genes would result in reduced activation. The hypotheses that *Ascl1* regulates deep states of quiescence and *Ascl1* induces *Mycn* expression emerged through data analysis and were not prespecified hypotheses. For immunohistochemical studies of conditional *Myc* knockout mice, sample sizes were determined based on previous studies that detected moderate to large effect sizes, such as our analysis of *Huwe1* cKO mice (*7*). For scRNA-seq analyses across different knockout strains, we performed at least two independent experiments to ensure reproducibility and to enable pseudo-bulk differential gene expression analysis, which is less error prone (*39*). Statistical testing of cell proportions was performed for the *Ascl1* cKO scRNA-seq dataset, as this experiment had sufficient statistical power due to the known larger effect size (*10*) and three independent replicates, compared to two replicates for *Huwe1* and *Mycn* cKO mice.

### Experimental models

All mice were maintained on a mixed genetic background. The *Ascl1* cKO mouse line with the neomycin cassette removed (*10*), *Myc* cKO (*21*) (MGI:2178233), *Mycn* cKO (*22*) (MGI:2388717), *Mycl* cKO (*23*) (MGI:4818995), Nestin–green fluorescent protein (GFP) (*40*) (MGI:5523870), YFP (*12*) (MGI:2449041), and Glast-creERT2 (*13*) (MGI:3830051) transgenic lines have all been previously reported. Both male and female mice were used throughout the study, an exception to this was for the x-linked Huwe1 conditional allele (*41*) (MGI:4439480), where only males were used. The effects of the gene knockouts seen throughout the study were consistent across sexes. All experimental protocols involving mice were performed in accordance with guidelines of the Francis Crick Institute, as well as national guidelines and laws. This study was approved by the UK Home Office (PPL PB04755CC). Throughout the study, mice were housed in standard cages with a 12-hour light/dark cycle and ad libitum access to food and water.

#### Preparation and sectioning of mouse brain tissue

Mice were perfused transcardially with phosphate-buffered saline (PBS), followed by 4% paraformaldehyde (10 to 20 ml), and postfixed for 16 to 24 hours before long term storage in PBS with 0.02% sodium azide, at 4°C. Brains were sectioned in a coronal plane at 40 μm using a vibratome (Leica). The entire rostral-caudal extent of the hippocampus was collected in a 1-in-12 series.

#### Antibodies, immunofluorescence, and cell counts

At minimum, one series per mouse (five to six sections) was stained and analyzed per experiment. Free-floating sections underwent antigen retrieval in sodium citrate solution (10 mM, pH 6.0) for 10 min at 95°C. The retrieval time was reduced if the detection of endogenous YFP/GFP was required, as these proteins are heat sensitive and cannot be re-detected with commercially available antibodies against GFP (*42*). Sections were blocked with normal donkey serum (2%) diluted in PBS–Triton X-100 (0.2%) for a minimum of 2 hours. Sections were then incubated overnight at 4°C with primary antibodies diluted in blocking buffer. After three washes in PBS for 10 min, the sections were incubated with fluorescent secondary antibodies at room temperature for 2 hours (Jackson ImmunoResearch). The sections were counterstained with 4′,6-diamidino-2-phenylindole (DAPI; Thermo Fisher Scientific) and mounted onto Superfrost slides.

Primary antibodies used for immunofluorescence in this study were rat monoclonal anti–glial fibrillary acidic protein (GFAP; Invitrogen, RRID:AB_86543, 1:800), chicken polyclonal anti-GFP (Abcam, RRID:AB_300798, 1:2000), mouse monoclonal anti-Ki67 (BD Biosciences, RRID:AB_393778, 1:100), rat monoclonal anti-SOX2 (Invitrogen, RRID:AB_11219471, 1:400), goat polyclonal anti-DCX (Santa Cruz Biotechnology, RRID:AB_2088494, 1:50), and rabbit monoclonal anti-MYCN (Cell Signaling Technology, RRID:AB_2800038, 1:200). Secondary antibodies were F(ab′)2 from Jackson ImmunoResearch raised in donkey (1:500) conjugated to 488, Cy3, or 647.

The sections were imaged using a SP5/SP8 Leica confocal microscope with a 40× objective or an Olympus CSU-W1 SoRa spinning disc confocal using a 20× objective. Cell counts were then performed, and data were normalized to number of cells per surface area of subgranular zone (SGZ), which was measured by the length of the SGZ in coronal brain sections multiplied by the thickness of the vibratome section. In all analyses, we identified NSCs as those cells that had a radial GFAP-positive process that we could confidently link to a SOX2-positive nucleus in the SGZ. The cells were classified as quiescent or proliferating depending on the expression of Ki67. To calculate the proportion of NSCs expressing MYCN in control mice, at minimum, 20 quiescent and proliferating NSCs were examined per animal. To calculate the proportion of NSCs expressing MYCN in *Ascl1* cKO versus *Ascl1* control mice, a minimum of 40 cells were examined per animal. Cell counts were not performed blinded to genotype, as the severe phenotypes of *Ascl1*, *Huwe1*, *Mycn*, and triple *Myc* cKO mice made blinding of images impractical.

#### Tamoxifen treatment

Tamoxifen solution (10 to 20 mg/ml) was prepared for oral gavage by dissolving the powder (Sigma-Aldrich) in a mix of 10% ethanol and 90% cornflower oil (Sigma-Aldrich) and was provided to mice via oral gavage (100 mg/kg).

### Single-cell RNA sequencing

#### Sample preparation

Each independent scRNA-seq experiment (batch) comprised a pooled group of one to two cKO mice (of either *Ascl1*, *Huwe1*, or *Mycn cKO* genotype) with one to two littermate controls that were prepared simultaneously to minimize processing artefacts. In total, three independent experiments were performed on *Ascl1* cKO mice and controls, two independent experiments on *Huwe1* cKO mice and controls, and two independent experiments on *Mycn* cKO mice and controls (table S1). In the *Ascl1* experiments, the cKO mice were homozygous for the floxed allele, heterozygous for Glast-creERT2, and homozygous for the cre-reporter YFP allele, while the control mice were genetically identical but were wild type for *Ascl1* alleles. In the *Huwe1* experiments, the male cKO mice were hemizygous for the floxed allele, heterozygous for Glast-creERT2, and homozygous for the cre-reporter YFP allele, while the male control mice were genetically identical but were wild type for *Huwe1*. All mice received tamoxifen via oral gavage (100 mg/kg) for 5 days starting from postnatal day 30 (P30; range from P27 to P33) and were euthanized 12 days later. For the *Mycn* experiments, the cKO and control mice were homozygous for the floxed allele, heterozygous for Glast-creERT2, and heterozygous for Nestin-GFP. The *Mycn* cKO mice received tamoxifen and *Mycn* control mice received corn-oil via oral gavage for 5 days at P30 (range from P27 to P30) before being euthanized 12 days later. In most experiments, at least two cKO or control mice were combined to guarantee the sorting and sequencing of sufficient numbers of cells (table S1).

Mice were euthanized by cervical dislocation, and the hippocampal dentate gyri were dissected (*43*, *44*). The dentate gyrus was disassociated using the Neural Tissue dissociation kit (P) (Miltenyi Biotec) with the following exceptions: A 37°C orbital shaker was used during the enzymatic digestions, and we used manual trituration with fire-polished pipettes to aid dissociation following the incubations with enzymatic mix 1 and 2 (*7*). Cells were also live-dead counterstained with DAPI. The cells were then sorted on a MoFlo XDP (Bechman Coulter) using a 100-μm nozzle. Debris were removed, followed by two gates to remove aggregates and dead cells, based on DAPI fluorescence. Cells were then gated for YFP or GFP expression according to cells from a control mouse hippocampus that did not contain a fluorescent transgene. We sorted between 10,000 and 25,000 cells per group (1 to 2 mice per group) over a period of 15 to 30 min per sample. The cells were sorted into 700 μl of recovery medium [0.5% PBS–bovine serum albumin in Dulbecco’s modified Eagle’s medium (DMEM)/F-12 without phenol red and HEPES, with L-glutamine] in 1.5-ml tubes and spun down at 500*g* for 7 min at 4°C. The cells were then gently resuspended in the residual recovery medium using a wide-bore pipette to a final volume of 50 μl. The single-cell suspension (to a maximum of 10,000 cells) was then loaded into the 10x Chromium.

#### Sequencing and data processing

On each experimental day, two libraries were prepared, one for each of the experimental groups to control for batch effects, which are strong in this type of data (i.e., cKO and control). All libraries were prepared with 10x Genomics Chemistry, Single Cell version 3.0.1. After sequencing, cellranger count (version 7.0.1) was used to map the FASTQ files from the Ascl1 and Huwe1 experiment to refdata-gex-mm10-2020-A. In contrast, the Mycn data were mapped to a custom version of this genome, which had the *Mycn* gene deleted and replaced with two contigs, corresponding to exon 1 of the *Mycn* gene (*Mycnex1*) and exon 2 and 3 (*Mycnex3*). This modification was made to detect cells that had undergone recombination of the *Mycn* locus. Specifically, in control cells, most of reads map to the exon 2 and 3 of the *Mycn* locus, which are directly upstream of the polyA tail. In contrast, in cells that have recombined *Mycn*, exons 2 and 3 are deleted, and all reads map to exon 1. The gene encoding enhanced GFP was also added to the custom genome. The genome was made with the cellranger mkref command. As both males and female mice were used in this study, but sex differences were not the focus of our investigation, the Y chromosome genes with highest expression (*Eif2s3y*, *Ddx3y*, *Gm47283*, *Uty*, and *Kdm5d*) and genes involved in X-inactivation (*Xist* and *Tsix*) were deleted from the count matrices before normalization and clustering.

#### Quality control

Individual samples were read into R version 4.2.0 and analyzed using Seurat (version 4.3.0.1) (*45*, *46*). Unless otherwise specified, plots were created using ggplot2 version 3.4.2. Gel Beads in Emulsion were excluded if the nCount_RNA values were less than 600 to 5000 (varied according to distribution of data within individual samples) or had greater than 8% mitochondrial reads (uniform across all samples). In total, 77.9% of cells passed quality control in Ascl1 data, 78.8% in Huwe1 data, and 94.9% in Mycn data. In the second *Ascl1* cKO replicate (comprising one male and one female), we found that a female mouse from the control group (compromising one male and one female) had been inadvertently swapped with the female *Ascl1* cKO mouse. Cells from the *Ascl1* cKO and control samples were reassigned to their correct experimental groups by first identifying the individual mouse each cell originated from on the basis of sex-specific gene expression. To do this, a sex score was calculated by subtracting raw counts of the sum of female genes involved in X-inactivation (*Xist* and *Tsix*) from Y chromosome genes (*Ddx3y*, *Uty*, and *Eif2s3y*). A negative sex score indicated female sex, and a score of ≥0 indicated male sex. Subsequently, the cells were assigned to the appropriate genotype by confirming the presence or absence of *Ascl1* expression.

#### Normalization, dimensionality reduction, and clustering

All *Ascl1* cKO and control samples were merged into one large Seurat object. Separately, this was repeated for all *Huwe1* samples and all *Mycn* samples. The merged Seurat objects were then split according to experimental replicate using the function SplitObject, and the data were transformed using the SCTransform function, with the vst.flavor argument set to “v2.” The data were integrated using the function SelectIntegrationFeatures with the nFeatures argument set to “3000” and the functions PrepSCTIntegration, FindIntegrationAnchors, and IntegrateData using default parameters to control for batch effects between experimental days. Elbow plots were used to approximate the number of principal components for clustering and visualization using Uniform Manifold Approximation and Projection (UMAP) (*47*). As axis values of UMAPs are not meaningful, we orientated the final plots so that proliferating NSC clusters appear toward the top-right of each plot and/or differentiation processes proceed from left to right.

After identification of clusters, we isolated the three neurogenic clusters according to known marker expression, quiescent NSCs (*Hopx*-high, *S100b*-low), proliferating cells (containing cell-cycle markers, NSC markers, and IPC markers), and neuroblasts (*Dcx*-high, *Eomes*-low). After isolating these cells, we used the SCTransform workflow to re-cluster these populations. Next, we sought to isolate only NSCs. As previously described, the proliferating cell cluster contained both proliferating NSCs and intermediate progenitor cells (*7*). Therefore, we next separated proliferating NSCs from intermediate progenitor cells based on marker expression (*Hopx/Apoe-*high, *Dcx/Neurod2-*negative; *Eomes/Tubb3*-low) and reiterated the SCTransform workflow. We repeated this process by removing proliferating NSCs and re-clustered a final time. The result was four Seurat objects (all cells, neurogenic clusters, NSCs, and quiescent NSCs) per *Ascl1*, *Huwe1*, and *Mycn* dataset. For downstream trajectory inference and pseudo-bulk analysis, Seurat objects were converted to SingleCellExperiment objects using the Seurat function as.SingleCellExperiment with the assay argument set to “RNA.”

#### Pseudo-bulk differential expression analysis

A pseudo-bulk approach was used to perform differential gene expression analysis between cKO and control conditions. Pseudo-bulk samples were created by aggregating counts across individual samples using the scuttle (version 1.8.4) function aggregateAcrossCells. Differential expression analysis was performed using the scran (version 1.26.2) function pseudoBulkDGE, which is a wrapper for edgeR’s quasi-likelihood methods. To run pseudoBulkDGE, the “edgeR” method was used, and batch effects between biological replicates (experiments) were accounted for using the design formula “~batch + genotype,” where batch represented the biological replicate and genotype represented cKO or control. Differentially expressed genes were determined using FDR < 0.05. Transcription factor status (0 and 1) of each gene was determined on the basis of a previously published classification (*48*). Down-regulated genes in *Ascl1* and *Mycn* cKO mice (FDR < 0.05, log fold change < 0) were used as features in Seurat’s AddModuleScore function to determine module scores.

#### GO enrichment analysis

GO enrichment analysis was performed using clusterProfiler (version 4.6.2) (*49*). First, gene symbols for differentially expressed genes identified by pseudo-bulk analysis were converted to Entrez IDs using the bitr function. The Entrez IDs were then passed to the enrichGO function with ont argument set to “BP” (biological process category). Significant terms were determined using FDR < 0.05.

#### Pseudotime analysis

For pseudotime analysis, the quiescent NSC Seurat objects were used. The quiescent NSCs were ordered from deep to shallow quiescence using the pseudotime inference tool Slingshot version 2.6.0 (*17*). Specifically, the slingshot function was used with reducedDim argument set to principal components analysis (PCA). To ensure that the orientation of the trajectory was correct, we assessed the expression of quiescence marker genes (*Apoe*, *Clu*, *Hopx*, *Id4*, and *Sparc*) and activation marker genes (*Ccnd1*, *Ccnd2*, *Rpl10*, and *Thrsp*), where we expect increased expression of the markers at the start and end of the trajectory, respectively. While Slingshot automatically placed the trajectory for the *Mycn* and *Huwe1* samples in the correct orientation, the trajectory for the *Ascl1* samples was in the reverse orientation. To overcome this, we created manual cluster labels based on Seurat clusters, where all but the Seurat cluster that should be the end cluster was labeled as one cluster. These manual cluster annotations were then used for the clusterLabels argument to run Slingshot.

#### Trajectory-based differential expression analysis

Downstream of trajectory inference, we performed trajectory-based differential expression analysis between experimental groups (cKO versus control) using tradeSeq version 1.12.0 (*25*, *26*). First, we evaluated the number of knots to use for the negative binomial generalized additive models (NB-GAMs) with the evaluateK function using default settings. On the basis of the graphical output from evaluateK, we purposely chose the same number of knots (six knots) across the *Ascl1* and *Mycn* experiments to enable comparisons. Next, we ran the fitGAM function to fit NB-GAMs to the SingleCellExperiment data, with nknots = 6 and conditions argument specifying genotype (cKO and control). Knots were visualized in reduced dimensions (PCA) using plotGeneCount. We then ran the conditionTest function multiple times to test for differential expression between experimental groups along pseudotime. For conditionTest, we used l2fc value of log_2_(1.2), as we found hundreds to thousands of significant genes without such a threshold, and, for each test, we contrasted sequential knots using the knots argument (vector of two), namely, knots 1 versus 2, 2 versus 3, 3 versus 4, 4 versus 5, and 5 versus 6. Results were sorted by descending Wald statistic, and *P* values were adjusted using p.adjust “fdr” method. Significant genes were determined using FDR < 0.05 and visualized using plotSmoothers.

#### ChIP-seq analysis

The ASCL1 ChIP-seq fastq file (ERR376162) was obtained from Andersen and colleagues (*10*) (deposited in the European Nucleotide Archive’s Sequence Read Archive under accession number ERP004380), and the p300 (ERR216112) and H3K27ac (ERR216108) ChIP-seq fastq files were obtained from Martynoga and colleagues (*27*) (deposited in the European Nucleotide Archive’s Sequence Read Archive under accession number ERP002084).

Raw reads data were quality controlled using FastQC (version 0.11.8). Reads were aligned using Bowtie2 (version 2.5.1) (*50*) to the mm10 mouse reference genome using default parameters. SAMtools (version 1.18) was then used to convert SAM files to BAM format. Sambamba (version 0.6.6) (*51*) was used to sort bam files by genomic coordinates and filter out polymerase chain reaction (PCR) duplicates and unmapped reads to contain only uniquely mapping reads. deepTools (version 3.5.1) (*52*) was used to obtain the bigWig files.

#### CUT&RUN

Wild-type mouse active AHNSCs were cultured in DMEM/F-12 and GlutaMAX (Invitrogen, 31331-093) medium supplemented with 1× N2 (STEMCELL Technologies, 05701), 1× penicillin/streptomycin (Thermo Fisher Scientific, 15140), laminin (3 μg/ml; Sigma-Aldrich, L2020), heparin (5 μg/ml; Sigma-Aldrich, H3393), and fibroblast growth factor (20 ng/ml; PeproTech, 450-33). Active AHNSCs were kept in incubator at 37°C in a 5% CO_2_ atmosphere.

The ASCL1 CUT&RUN samples (two replicates) were performed with the CUT&RUN kit (CUTANA ChIC/CUT&RUN EpiCypher). Briefly, 5 × 10^5^ cultured AHNSCs were collected in a 1.5-ml tube containing wash buffer. The cells were captured with ConA beads and incubated with 0.5 μg of the anti-ASCL1 monoclonal antibody (Abcam, RRID:AB_2924270) overnight at 4°C in antibody buffer. After washing the unbound antibody, Protein A/G Micrococcal Nuclease was added and incubated for 10 min at room temperature. After washing, CaCl_2_ was added and incubated for 2 hours at 4°C, and the reaction was stopped with the stop buffer. The protein-DNA complexes were released by incubating at 37°C for 10 min. DNA was then purified using a DNA cleanup kit. Sequencing library was prepared using the NEBNext Ultra II DNA library preparation kit for Illumina (New England Biolabs) and following the described protocol (*53*, *54*) specifically modified to make libraries from small DNA fragments. Briefly, end repair was conducted at 20°C for 30 min, followed by deoxyadenosine monophosphate nucleotide tailing at 50°C for 60 min. After adapter ligation, the DNA fragments were purified by 1.75× volume of AMPure XP beads (Beckman Coulter; A63881) followed by 14-cycle PCR amplification with NEBNext Ultra II Q5 Master Mix (New England Biolabs, E7645L). The PCR products were firstly cleaned up with 0.8× volume of AMPure XP beads. Then, a second cleaning was performed with 1.2× volume of AMPure XP beads to remove PCR products shorter than 150 bp. A final round of 1.2× AMPure XP beads clean-up was performed to eliminate PCR dimers. The CUT&RUN library was quantified using the Qubit fluorometric quantification system, and quality control was performed with the Bioanalyzer High Sensitivity DNA Analysis (Agilent). The library was sequenced on the NovaSeq 6000 instrument, PE100. For the analysis of the CUT&RUN data, the nf-core/cutandrun pipeline was used (version 3.2) (*55*). The uniquely mapped reads were aligned to the mm10 reference genome using Bowtie2 (*50*). Peak calling was performed using Sparse Enrichment Analysis for CUT&RUN (SEACR) with 0.01 seacr_peak_threshold (*56*). The BigWig file was visualized using the Integrative Genomics Viewer.

### Statistical analysis of cell counts

The statistical testing approach was implemented using GraphPad Prism (version 8.0) or in R (4.2.0). Two-tailed unpaired Student’s *t* tests were performed when comparing two groups. For experiments involving two independent variables, a two-way analysis of variance (ANOVA) was performed. Any significant main effect detected by ANOVA was followed by multiple comparisons analysis using multiple *t* test and a pooled estimate of variance where appropriate with Holm-Šídák correction. Individual values for sample sizes of <20 are found in table S5.

### Statistical analysis of single-cell data

To determine whether the distribution of quiescent NSCs along pseudotime was different between experimental groups, a Kolmogorov-Smirnov test was performed with function ks.test in R (4.2.0).
