# Supplementary material for: Sequential transcriptional programs underpin activation of hippocampal stem cells
Source: Sci Adv. 2025 Jun 11;11(24):eadu4523. doi: 10.1126/sciadv.adu4523 (PMC12154177; doi:10.1126/sciadv.adu4523)
Supplement: Supplementary file 1 — Figs. S1 to S4 Legends for tables S1 to S5 [file sciadv.adu4523_sm.pdf]

Science Advances
AAAS

# Supplementary Materials for

## Sequential transcriptional programs underpin activation of hippocampal stem cells

Piero Rigo *et al.*

Corresponding author: Lachlan Harris, lachlan.harris@qimrb.edu.au

*Sci. Adv.* **11**, eadu4523 (2025)
DOI: 10.1126/sciadv.adu4523

**The PDF file includes:**

Figs. S1 to S4
Legends for tables S1 to S5

**Other Supplementary Material for this manuscript includes the following:**

Tables S1 to S5

Figure S1

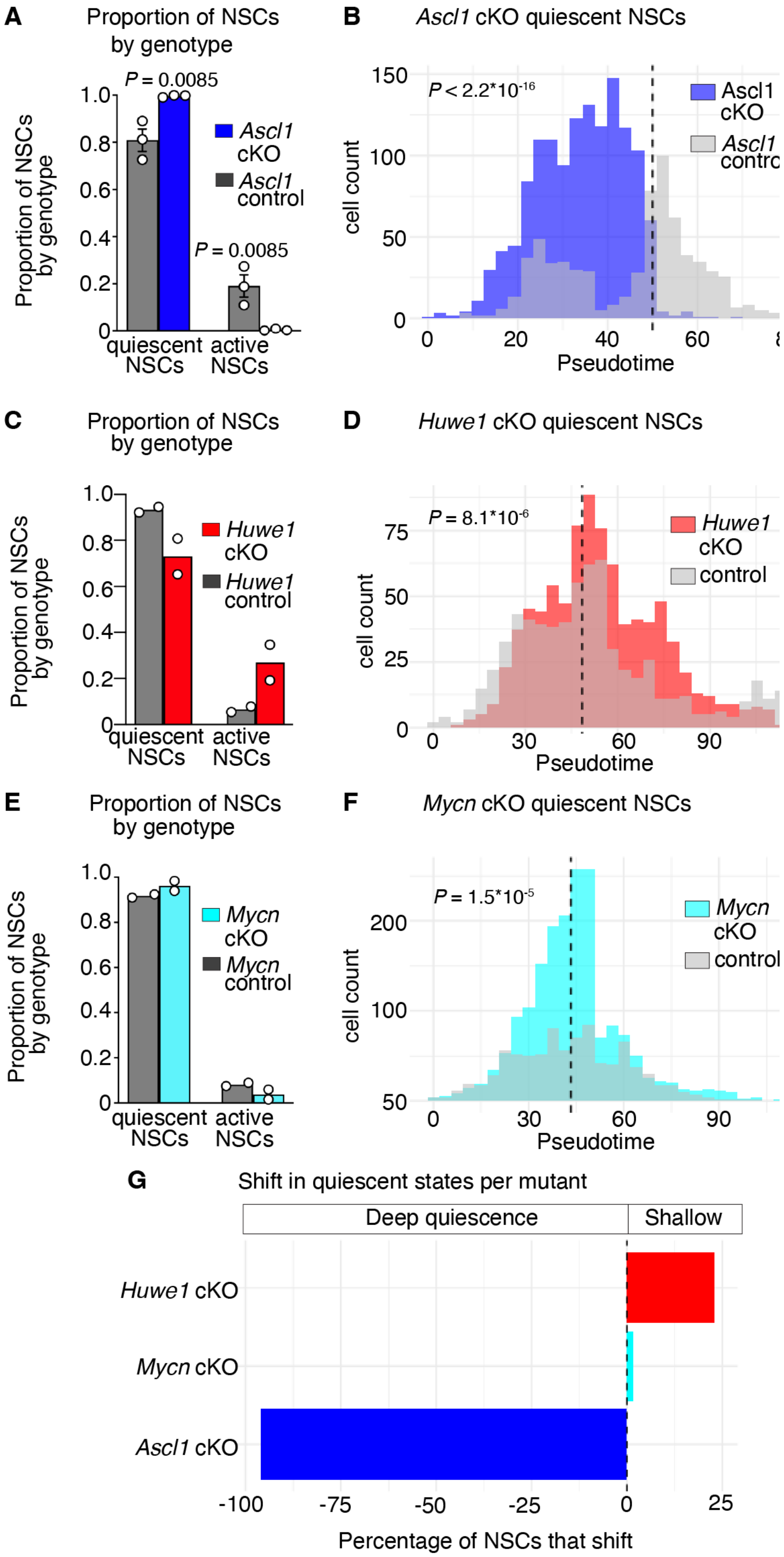

A
Proportion of NSCs by genotype
P = 0.0085
Ascl1 cKO
Ascl1 control
Proportion of NSCs by genotype
quiescent NSCs
active NSCs
B
Ascl1 cKO quiescent NSCs
P < 2.2*10-16
Ascl1 cKO
Ascl1 control
cell count
Pseudotime
C
Proportion of NSCs by genotype
Huwe1 cKO
Huwe1 control
D
Huwe1 cKO quiescent NSCs
P = 8.1*10-6
Huwe1 cKO
control
E
Proportion of NSCs by genotype
Mycn cKO
Mycn control
F
Mycn cKO quiescent NSCs
P = 1.5*10-5
Mycn cKO
control
G
Shift in quiescent states per mutant
Deep quiescence
Shallow
Huwe1 cKO
Mycn cKO
Ascl1 cKO
Percentage of NSCs that shift

**Fig. S1. Effect of *Ascl1*, *Mycn* and *Huwe1* loss on NSC quiescence, related to Figure 1, 2 and 5.**

(A) Quantification of quiescent and proliferating NSCs in *Ascl1* cKO and control mice. Dots indicate independent experiments.

(B) Histogram of quiescent NSCs from *Ascl1* cKO and control mice arranged from deep to shallow quiescence (number of bins = 30). Dashed line indicates median position of control NSCs. This is a different visualisation of the same data presented in Figure 1F (right).

(C) Quantification of quiescent and proliferating NSCs in *Huwe1* cKO and control mice. Dots indicate independent experiments.

(D) Histogram of quiescent NSCs from *Huwe1* cKO and control mice arranged from deep to shallow quiescence (number of bins = 30). Dashed line indicates median position of control NSCs. This is a different visualisation of the same data presented in Figure 2F (right).

(E) Quantification of quiescent and proliferating NSCs in *Mycn* cKO and control mice. Dots indicate independent experiments.

(F) Histogram of quiescent NSCs from *Mycn* cKO and control mice arranged from deep to shallow quiescence (number of bins = 30). Dashed line indicates median position of control NSCs. This is a different visualisation of the same data presented in Figure 5F (right).

(G) Quantification of the proportion of quiescent NSCs in cKO mice that shift towards deep quiescence (less than median pseudotime position of control cells) or towards shallow quiescence (greater than median pseudotime position of control cells).

Statistics: Multiple comparison t-test in (A) reporting Holm-Sidak corrected *P*-value; Kolmogorov-Smirnov test in (B, D and E).

Figure S2

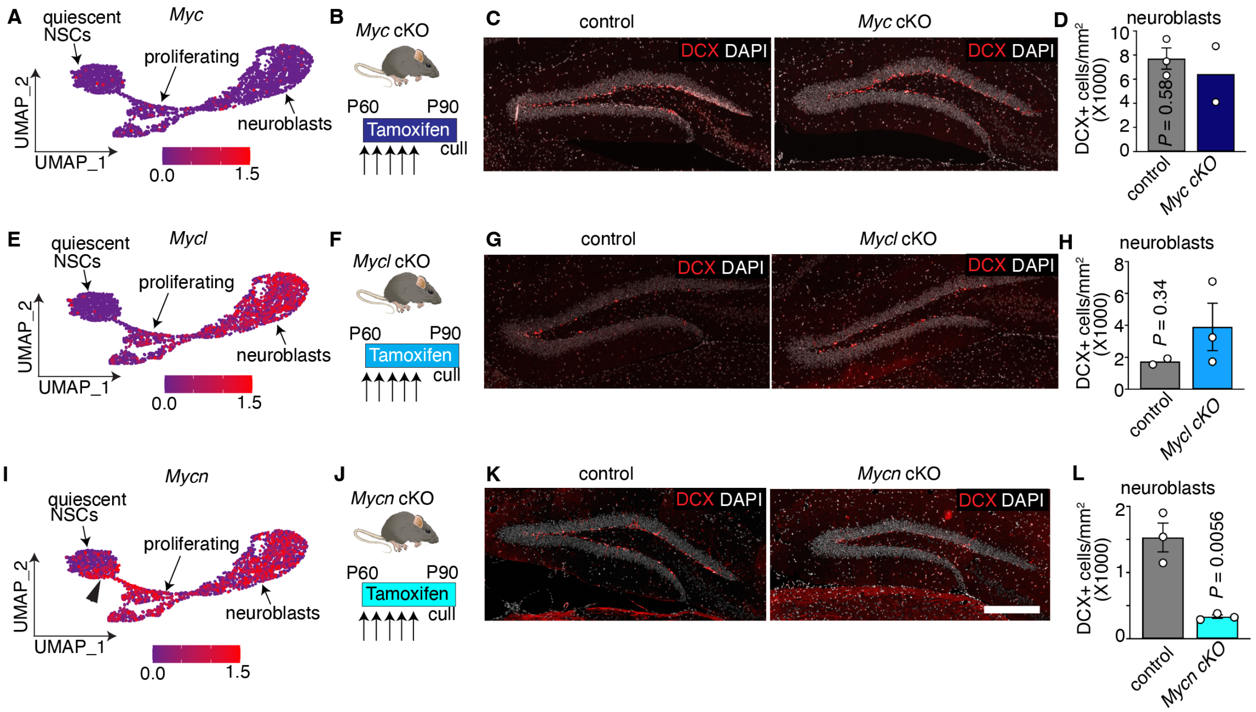


**Fig. S2. Loss of *Myc* or *Mycl* does not substantially impair adult hippocampal neurogenesis, related to Figure 3-5.**

(A) Expression of *Myc* in adult hippocampal neurogenic lineage, data from *Ascl1* control mice in Figure 1.

(B) *Myc* cKO and control mice were injected with tamoxifen for 5 days and culled 30 days later.

(C) DCX staining in *Myc* cKO mice was comparable to controls.

(D) Quantification of DCX+ cells in *Myc* cKO mice and controls. Dots indicate individual mice.

(E) Expression of *Mycl* in adult hippocampal neurogenic lineage, data from *Ascl1* control mice in Figure 1.

(F) *Mycl* cKO and control mice were injected with tamoxifen for 5 days and culled 30 days later.

(G) DCX staining in *Mycl* cKO mice was comparable to controls.

(H) Quantification of DCX+ cells in *Mycl* cKO mice and controls. Dots indicate individual mice.

(I) Expression of *Mycn* in adult hippocampal neurogenic lineage, data from *Ascl1* control mice in Figure 1.

(J) *Mycn* cKO and control mice were injected with tamoxifen for 5 days and culled 30 days later.

(K) DCX staining in *Mycn* cKO mice was substantially reduced compared to controls.

(L) Quantification of DCX+ cells in *Mycn* cKO mice and controls. Dots indicate individual mice.

Scale bar (located in K): 320 µm in (C, G, K). Arrowhead (located in I) indicates group of quiescent NSCs expressing *Mycn* but not *Myc* or *Mycl*.

Statistics: Unpaired t-test in (D, H, L)

## Figure S3

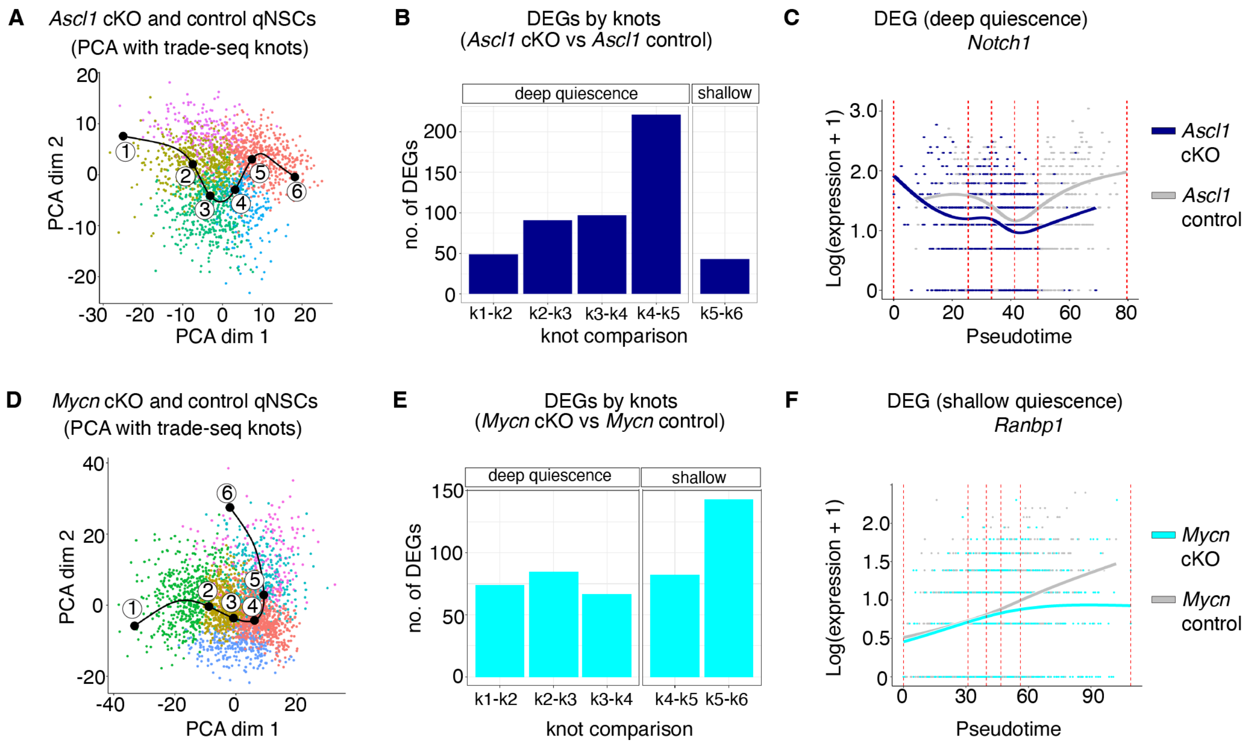


**Fig S3. *Ascl1* loss impacts deeper stages of NSC quiescence than *Mycn* loss, related to Figure 6.**

(A) Principal Component Analysis (PCA) dimensions 1 and 2 of *Ascl1* cKO and control quiescent NSCs, labelled with six knots marking binning of cells into five equally sized partitions of pseudotime as determined by tradeSeq. Cells coloured by Seurat cluster.

(B) Number of differentially expressed genes (DEGs) at each knot comparison between *Ascl1* cKO and control quiescent NSCs. Deep quiescence and shallow quiescence defined as knots that occur before and after median pseudotime position of control cells, respectively.

(C) Example of a DEG from early knot comparisons (deep quiescence) between *Ascl1* cKO and control quiescent NSCs.

(D) PCA dimensions 1 and 2 of *Mycn* cKO and control quiescent NSCs, labelled with six knots marking binning of cells into five equally sized partitions as determined by tradeSeq. Cells coloured by Seurat clusters.

(E) Number of DEGs at each knot comparison in *Mycn* cKO and control quiescent NSCs. Deep quiescence and shallow quiescence defined as knots that occur before and after median pseudotime position of control cells, respectively.

(F) Example of a differentially expressed gene from late knot comparisons (shallow quiescence) between *Mycn* cKO and control quiescent NSCs

## Figure S4

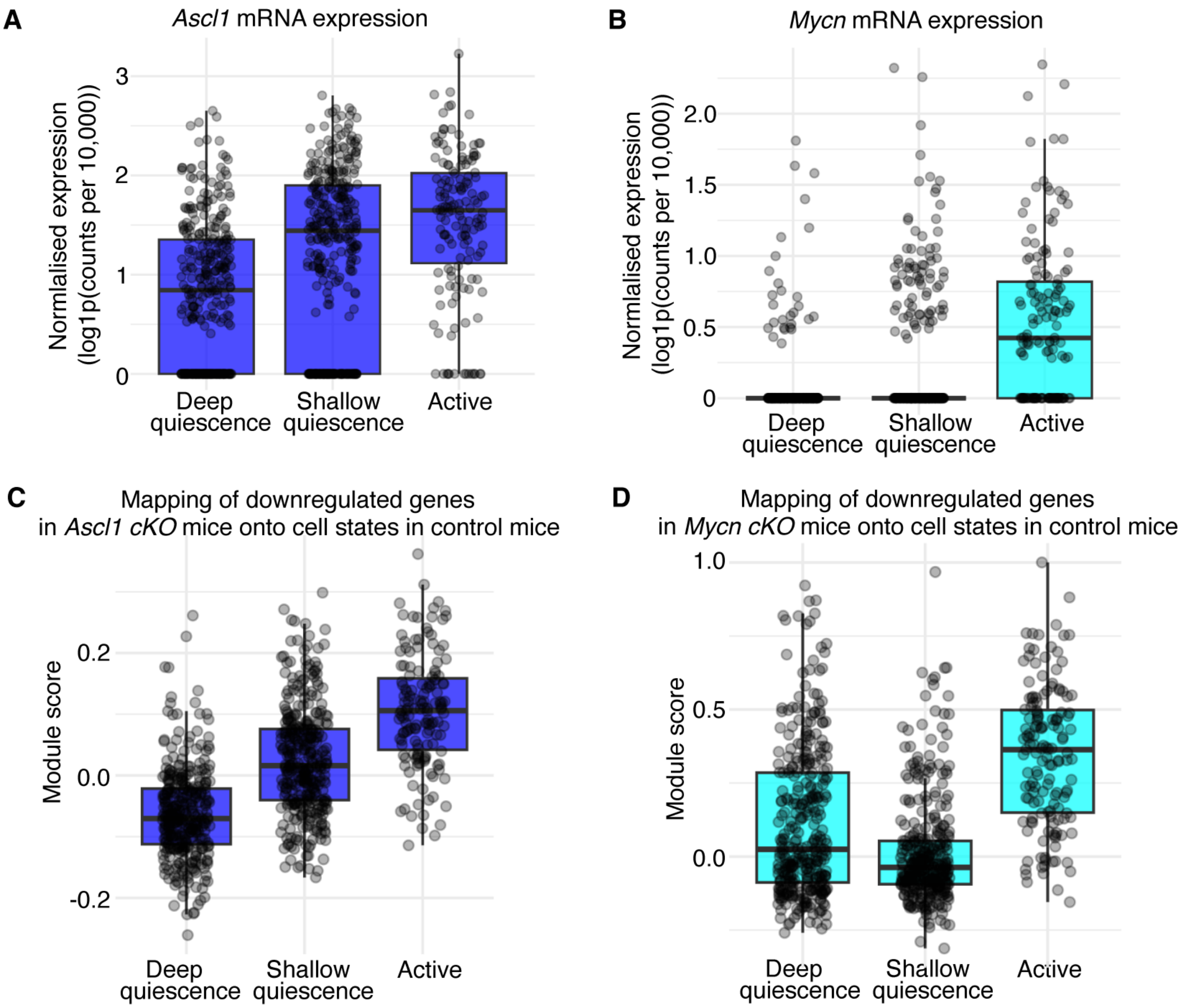


**Fig S4. A sequential *Ascl1*-*Mycn* program drives NSC activation, related to Figure 6.**

(A) *Ascl1* mRNA expression in deep, shallow and proliferating NSC states. Data from *Ascl1* control mice in Figure 1.

(B) *Mycn* mRNA expression in deep, shallow and proliferating NSC states. Data from *Ascl1* control mice in Figure 1.

(C) Module score of downregulated genes in quiescent NSCs from *Ascl1* cKO mice (Table S2) mapped onto cell states in *Ascl1* control mice from Figure 1.

(D) Module score of downregulated genes in quiescent NSCs from *Mycn* cKO mice (Table S4) mapped onto cell states in *Ascl1* control mice from Figure 1.

**Table S1. (separate file)**
List of scRNA-seq experiments.

**Table S2. (separate file)**
Differential expression analysis of *Ascl1* cKO NSCs versus control NSCs, and differential expression analysis and gene ontology analysis of *Ascl1* cKO quiescent NSCs versus control quiescent NSCs.

**Table S3. (separate file)**
Differential expression and gene ontology analysis of *Huwe1* cKO NSCs versus control NSCs.

**Table S4. (separate file)**
Differential expression and gene ontology analysis of *Mycn* cKO quiescent NSCs versus control quiescent NSCs.

**Table S5. (separate file)**
Quantitative data for comparisons with N < 20.
